# Anterior Cruciate Ligament Reconstruction with LARS Synthetic Ligament: Outcomes and Failures

**DOI:** 10.3390/jcm14010032

**Published:** 2024-12-25

**Authors:** Lorenzo Moretti, Raffaele Garofalo, Giuseppe D. Cassano, Alessandro Geronimo, Nicola Reggente, Fabrizio Piacquadio, Davide Bizzoca, Giuseppe Solarino

**Affiliations:** 1 Orthopaedic & Trauma Unit, Department of Basic Medical Sciences, Neuroscience and Sense Organs, School of Medicine, University of Bari Aldo Moro, AOU Consorziale Policlinico, 70124 Bari, Italy; 2 Department of Orthopaedics and Traumatology, Ente Ecclesiastico Ospedale “F. Miulli”, Acquaviva Delle Fonti, 70021 Bari, Italy

**Keywords:** knee, anterior cruciate ligament, ACL, ACL reconstruction, synthetic ligaments, LARS ligament

## Abstract

**Background:** Anterior cruciate ligament (ACL) injuries are common in athletes, but their prevalence has also increased among adults. ACL reconstruction (ACLR) is a key treatment option, with graft choice playing a critical role in recovery. The study evaluates the clinical and functional outcomes of ACLR using the Ligament Augmentation and Reconstruction System (LARS) in patients over 35 years old. It assesses implant survival, failure rate, and revision rate, and measures quality of life and subjective outcomes. **Methods**: Fifty-three patients were finally included in this retrospective two-center study. The study assessed quality of life (QoL) and subjective outcomes using IKDC and Lysholm scores, evaluated implant survival and revision rates, and assessed the difference in activity levels between the two years before ACL injury and at follow-up. **Results:** The study found high Lysholm (90.61) and IKDC (80.25) scores, indicating positive clinical results. However, about 40% of patients did not return to their pre-injury activity levels. The graft failure rate was low at 3.8%, with no cases of infection or implant rejection. **Conclusions:** The study concluded that LARS ACLR is an effective option for middle-aged patients, offering faster recovery and fewer complications. However, it may not be suitable for younger, professional athletes due to its mechanical limitations. Further research with larger sample sizes and longer follow-up is recommended.

## 1. Introduction

Anterior cruciate ligament (ACL) tears affect a significant number of young athletes. However, as sports become more popular, ACL injuries are also increasing in adults [1,2]. Anterior cruciate ligament reconstruction (ACLR) is a widely used treatment for active individuals with ACL injuries, aiming to restore knee joint stability and function. There are different graft choices and different techniques for ACLR [3]. Graft choice plays a very important role, especially when we talk about athletes who want to return to sports as soon as possible or when we are faced with middle-aged patients [4,5]. The graft used for ACLR could be an autograft or an allograft from human cadavers, a xenograft from animals, or a synthetic graft [6]. Autografts are used more frequently and show good results in terms of surgical results and the ability to return to sports, even if a growing number of alternative choices for grafts are becoming available for orthopedic surgeons, such as synthetic grafts [7].

High failure rates, sterile effusions from wear particles, and foreign body synovitis have limited the use of synthetic grafts [8,9]. Over the last few decades, several synthetic grafts have been produced and marketed. However, preliminary results for newer-generation devices, specifically the Ligament Augmentation and Reconstruction System (LARS) composed of terephthalic polyethylene polyester fibers, show lower reported rates of failure, revision, and sterile effusion/synovitis when compared with older devices [10]. The LARS consists of an extra-articular and an intra-articular part. The scaffold or intra-articular portion of the ligament consists of several parallel fibers twisted at a 90° angle. This component is designed following the anatomic structure of the ACL and is made up of two longitudinal external rotation fibers without transverse fibers. To avoid ligament distortion, longitudinal and transverse fibers are employed to make waves on the extra-articular portion. However, it is important to remember that osseointegration is partial, so their retention is mainly ensured by primary fixation [11,12,13]. In addition, the risk of rupture increases proportionally with time after surgery, while the strength of the ligament decreases over time [14]. Synthetic grafts have been used in most cases, mainly in patients over 40 years old or with low functional demands. On the other hand, there are several articles that evaluate their use, mainly in patients over 40 years old, ensuring a rapid functional recovery and a rapid return to daily activities and sports [15]. In addition, some authors highlighted a low rate of progression of osteoarthritis over time at a follow-up of 5 years [15]. A previous study we published showed no differences between the groups in anterior tibial translation (ATT) values and clinical outcomes, as assessed by the Lysholm score when comparing ACLR with a synthetic graft to quadrupled semitendinosus graft in patients over 30 years old [16].

Although the literature lacks studies regarding the outcomes of patients undergoing ACLR with LARS, the few data reported in previous scientific publications tell us that in the short term, the results are very satisfactory, with a faster recovery compared to traditional autografts. Some systematic reviews have highlighted the effectiveness of ACLR with LARS with comparable results, and in some publications, the results are even better than autograft techniques in the short term. However, there is little evidence reported in the long term, although there appear to be no significant differences between the two groups [17,18].

This was confirmed by a recent meta-analysis published in 2020, which underlined that ACLR with LARS produced better postoperative outcomes in terms of restoration of knee joint function and stability and was associated with fewer postoperative complications. In cases where early rehabilitation is critical, multiple ligament injuries are present, or autologous tissue is not easily available for reconstruction, the authors suggest the LARS ligament to be a viable alternative for ACLR [19].

The evidence reported in the studies mentioned above is more than a decade old. It would be useful to have a study with a large sample, including two centers and with a long follow-up. This study aims to evaluate the clinical and functional outcomes of patients undergoing ACLR with the LARS synthetic ligament and the potential rate of graft failure over time. The study will enroll a significant number of patients with ACL injuries and assess their long-term outcomes, including clinical–functional and return to sport outcomes. The trial lacks a control group of patients who have undergone ACLR with autograft, but scientific evidence from the literature will help to compare outcomes. The ultimate goal is to assess whether patients undergoing ACLR with the LARS achieve good clinical outcomes, avoid high complications, and return to sports activity at a comparable level to pre-injury.

## 2. Materials and Methods

### 2.1. Study Design and Sample

This is an observational, retrospective, two-center study. The study was approved by the local ethical committee (protocol number: 12/CE/2022—1 May 2022) by the University of the Study of Bari “Aldo Moro”; all patients gave informed consent prior to enrollment.

Inclusion criteria were patients undergoing ACLR with synthetic ligaments, aged over 35 years old. Exclusion criteria were combined multiple knee ligament injuries, ACL revision surgery, history of infection, lower limb coronal axial deviation > 10°, patients with chondral damage Outerbridge grade > 2, and patients with knee OA Kellgren–Lawrence (KL) grade > 3.

Seventy-two (72) patients undergoing ACLR with a synthetic ligament were identified in the database from January 2015. Nineteen patients (19) were excluded, of which seventeen (17) did not meet inclusion and exclusion criteria, and two (2) refused to participate in the study, leaving fifty-three (53) patients included. Forty-five (45) were men and eight (8) were women. Of these, 48 were recreational athletes and 5 were semi-professional. They were evaluated between January and May 2024. For each patient, the following data were recorded: age, sex, and time since surgery (rounding up from the first year).

**Figure 1:**
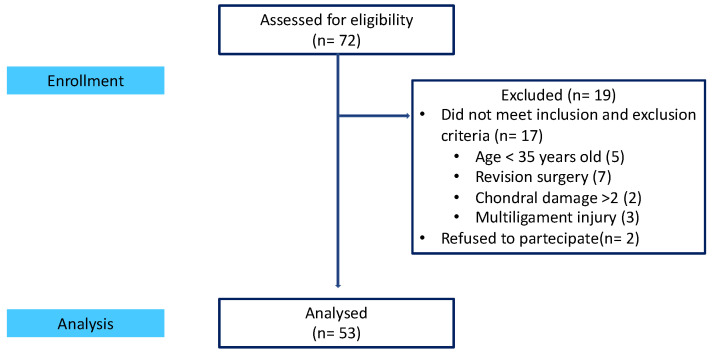
Study phases.

The flowchart of patient selection is shown in Figure 1.

### 2.2. Clinical Evaluation

The primary aim is to evaluate implant survival and failure rate or revision rate. After clinical evaluation, as the secondary endpoint, the Lysholm questionnaire and the International Knee Documentation Committee (IKDC) questionnaire have been used to evaluate quality of life (QoL) and subjective outcomes. The Lysholm Knee Scoring Scale is a patient-reported instrument that consists of subscales for pain, instability, locking, swelling, limping, stair climbing, squatting, and the need for support. Scores range from 0 (worst disability) to 100 (less disability). The IKDC is a patient-completed tool, which contains sections on knee symptoms (7 items), function (2 items), and sports activities (2 items). Scores range from 0 points (lowest level of function or highest level of symptoms) to 100 points (highest level of function and lowest level of symptoms).

As a tertiary endpoint, the difference in activity level according to the Tegner scale between the 2 years before the ACL injury and the activity level at follow-up was also assessed.

### 2.3. Surgical Technique

Surgery is performed under spinal anesthesia, with the patient placed in a supine position on the operating table. After Hemaclear surgical tourniquet (OHK Medical Devices, Inc. 2885 Sanford Ave. SW #14751 Grandville, MI 49418, USA) positioning and preparation of the leg, the arthroscopic portals were created. Following diagnostic arthroscopy, associated meniscal tears or chondral lesions were treated and partial notchplasty was performed.

A 110° femoral aimer (Femoral ACL Marking Hook for Retro-Construction Drill Guide-^®^—Arthrex©, Naples, FL, USA) and a 65° tibial aimer (Tibial ACL Marking Hook) were pointed at the anatomical ACL footprints under a direct arthroscopic view. The retrograde femoral half tunnel using a FlipCutter^®^ III Drill (Arthrex©, Naples, FL, USA) was created, and it measured about 2.5 cm. A complete tibial tunnel was created using cannulated drills on a Kirschner wire with progressive diameters up to 8 mm. The LARS synthetic ligament was duplicated and fixed on the femur side with a suspension system (Tight rope Arthrex). Tibial fixation should be completed with a non-absorbable metallic interference screw at least 1 mm larger in diameter than the tunnel.

### 2.4. Statistical Analysis

Data were collected and analyzed using Microsoft Excel and SPSS IBM 29.0. Categorical variables were presented as numbers or percentages. Continuous variables were presented as mean and standard deviation. All variables were tested for normality using the Shapiro–Wilk test. The Mann–Whitney U test was used when normality was rejected.

A *p*-value of <0.05 was considered statistically significant. The data presented in this study are available on request from the corresponding author.

## 3. Results

A total of 53 subjects were enrolled in this study (42.04 ± 4.55 mean age, 45 male, 40.42 ± 19.14 mean time evaluation after surgery months). The main demographic characteristics are described in Table 1.

Two patients (3.8%) showed implant failure with instability and formation of an osteolytic area around the interference screw at the tibial level. These patients underwent two-stage surgery, the first stage consisting of the removal of the synthetic ligament and screw and filling with autologous bone from the iliac crest in the tibial and femoral tunnels, and the second stage consisting of new ACL reconstruction with autologous grafting. Two patients (3.8%) presented synthetic ligament lesion, and two other patients (3.8%) underwent surgery for new meniscal tears. One patient (1.9%) underwent total knee arthroplasty for grade 3 KL arthrosis about 4 years after the ACLR. None of the patients showed signs of infection or implant rejection.

At follow-up, the main Lysholm score was 90.61 ± 10.66 and the main IKDC score was 80.25 ± 12.40.

In the 2 years before the injury, the main Tegner activity score was 6.08 ± 2.15, while at follow-up, it was 5.35 ± 2.02. The difference between the two times was statistically significant (*p* = 0.021). A total of 57% of patients returned to the same level of activity as before, 4% returned to more physically demanding activities according to the Tegner’s grade after surgery, while the remaining 39% did not return to the same type of sport.

## 4. Discussion

Reconstruction of the anterior cruciate ligament using autografts remains the gold standard to date. However, this method is a bit reminiscent of the “steal from Peter to pay Paul” theory. For this reason, the use of synthetic ligaments has become increasingly popular and brings with them a whole series of benefits: shorter surgical time, fewer postoperative complications, reduced morbidity at the harvest site, faster postoperative recovery, and lower incidence of postoperative arthrofibrosis [19].

The study we conducted confirmed this positive trend with excellent clinical outcomes, with an average Lysholm score over 90 and an IKDC score over 80, thus confirming the effectiveness and safety of the LARS-ACLR method. However, in terms of return to sport (RTS), we reported that almost 40% of operated patients did not return to the same sport, and with an average reduction in the Tegner activity scale of almost 1 point compared to two years before the injury.

This result (decrease) is similar to that obtained by Zhenyu Jia in a study with a 90-month follow-up. The Tegner score in the postoperative period was 4.7 ± 1.3, while the preoperative value was 5.5 ± 1.0. For the Lysholm score, the authors described an improvement from a preoperative value of 54.6 ± 14.3 to a postoperative value of 85.4 ± 12.1 [20].

An element that may have affected the RTS outcomes and Tegner activity level can be found in the fact that none of the patients practiced sports at a competitive or professional level; therefore, they did not follow an intense rehabilitation protocol or preferred to reduce the level of sporting activity. Furthermore, middle-aged patients, especially those who practice sports at an amateur and non-professional level, do not have the same physical performance and recovery as young players and are also less compliant and motivated in the phase following surgery.

As far as failures are concerned, the two cases of osteolysis could be due to a non-optimal bone quality or to a change in the position of the interference screw in the tunnel or to the activity of the patients, which first caused a partial mobilization of the implant, and then osteolysis due to the detection of repeated microtraumas. In these two cases, a two-stage reconstruction had to be performed. There were no proven cases of synovitis or intolerance of the implant.

Several studies in the literature confirm our results. In an old studio with a previous generation system, Dericks et al. conducted a mean 2.5-year follow-up and found no cases of knee synovitis in 220 cases [21]. Parchi et al. reported a positive outcome rate of 92% of patients at a mean follow-up of 8 years and reported only one complication (mechanical graft failure) in a sample of 26 patients. Most patients reported good satisfaction after ACLR with LARS: using the KOOS grading scale, a mean score of 84 was obtained, with 11 (42.3%) optimal results, 13 good results (50%), and two bad results (7.7%) [22]. In another paper with a short-term follow-up, the authors highlighted the restoration of anterior tibial translation and anterolateral rotational stability of the affected knee joint following ACLR with a synthetic ligament [23].

On the other hand, a 10-year postoperative follow-up study by Tiefenboeck et al. on 18 patients showed that 50% of patients treated with the LARS were not satisfied at all: 27.8% underwent revision surgery after a new graft failure. The authors recommended that synthetic grafts should only be used in certain situations and not as a primary option for ACLR [24].

Similar data have been reported by Smolle et al. on a total of 41 patients, with a complication rate of 66% (27 patients); 11 developed within one year following surgery and 16 developed after one year. The most frequent complication was the graft failure (24% of patients), while reactive synovitis was observed in 20% of the cases [25].

In our series, two patients (3.8%) presented with implant rupture. Regarding implant failure and rupture, Di Benedetto and colleagues tried to explain what this could be due to through histological evaluation. They evaluated that biologic issues (poor tissue ingrowth, the histological analysis of the removed LARS revealed a surrounding typical foreign body reaction with poor signs of fibrovascular ingrowth of the synthetic ligament) and mechanical issues (fibers properties and tunnel position) probably concur in a multi factorial manner [26]. We believe that a “killer angle”, i.e., a particularly acute angle of the femoral tunnel can promote early graft wear.

The primary benefits of this graft choice are the potential to place a greater load on the limb in less time compared to natural grafts, stronger mechanical strength, no problems related to graft harvesting, and no need to respect the biological times of ligamentization [27,28]. After implantation, synthetic ligaments do not need revascularization, so they can immediately have important mechanical effort.

However, because of their lack of viscoelasticity and propensity for abrasion, they are unable to reach the biomechanical properties of ACL [10].

Synthetic grafts were also more expensive than autologous grafts.

Middle-aged patients, especially those who practice sports at an amateur and non-professional level, do not have the same physical performance and recovery as young players and are also less compliant and motivated in the phase following surgery.

We believe that the surgical indication could be extended to people over 35 or over 40 who do not exercise regularly or whose exercise is limited. We do not believe that this technique is useful in professional athletes and even less so in young patients [29].

However, we have no experience with LARS augmentation in combination with ACLR with autografts, and we do not believe that this hybrid construct could be an adequate solution. However, there are studies in the literature. In fact, in a case series, Ebert et al. [30] analyzed 65 patients treated with arthroscopically assisted single-bundle ACLR using hamstrings augmented with the LARS.

The authors report that the diameter of the graft increased by just over 1 mm and demonstrated good clinical outcomes and high levels of satisfaction, including the ability to participate in sport [30]. Other authors have also shown satisfactory results with this hybrid technique [31]. In a recent comparative study between the hybrid and autograft techniques, Aujla and colleagues showed that augmentation ACLR patients had higher 1-year postoperative Tegner scores and rates of RTS and preoperative sport levels compared to the hamstring ACLR group. The 2-year re-rupture rate for the hybrid group was also low and no intra-articular inflammatory complications were noted [32].

What has been highlighted in our work is the confirmation of the initial hypotheses of the study objective in terms of clinical–functional outcomes, as demonstrated by the high IKDC (80.25) and Lysholm score (90.61) values at the 2-year follow-up, highlighting its potential as an option for improving quality of life in middle-aged adults. This was consistent with previous scientific findings [20]. Complication rates were also low. The only unsatisfactory aspect was the Tegner activity score; while the majority of patients experienced a successful recovery, 40% did not return to their pre-injury activity level, with patients reporting a decrease in their average level of sports activity, albeit in a moderately negative range. Our study has some weaknesses. Patients were enrolled for surgery in two different centers, and this could potentially cause some bias in the results. Other limitations of the study are the lack of information on the tibial slope, the lack of assessment of chondral and meniscal damage and the consequent statistical analysis, and no long-term follow-up.

## 5. Conclusions

This study shows that ACL reconstruction using LARS synthetic ligaments is effective for middle-aged patients, particularly those over 35 who are not professional athletes. At mid-term follow-up, clinical outcomes were positive, with high Lysholm and IKDC scores, but about 40% of patients did not return to their previous level of activity. The LARS offers benefits such as faster recovery and fewer complications, but its mechanical properties may limit its use in younger or professional athletes. The graft failure rate was low (3.8%) and there was no reported evidence of infection.

Limitations include the retrospective design and lack of analysis of tibial slope or associated meniscal and chondral lesions. Further studies with larger sample sizes and long-term follow-up are needed to assess the rate of graft failure.

## Figures and Tables

**Table 1:** Baseline evaluation of study participants.

Preoperative Features	Group A
Number of patients	53
Age	42.04 ± 4.55
Side (right)	23 right—30 left
Gender	45 male—8 women
Time from surgery (months)	40.42 ± 19.14

## Data Availability

The data presented in this study are available upon request from the corresponding author. The data are not publicly available due to privacy concerns.
